# Antipsychotic-Associated Galactorrhea May Mask Diffuse Ductal Carcinoma in Situ: A Diagnostic Challenge

**DOI:** 10.7759/cureus.99706

**Published:** 2025-12-20

**Authors:** Ilze Engele, Tatjana Zablocka, Evija Asere, Janis Eglitis

**Affiliations:** 1 Department of Radiology, Riga East University Hospital, Riga, LVA; 2 Faculty of Medicine and Life Sciences, University of Latvia, Riga, LVA; 3 Radiology, Institute of Clinical and Preventive Medicine, University of Latvia, Riga, LVA; 4 Department of Pathology, Riga East University Hospital, Riga, LVA; 5 Department of Breast Surgery, Riga East University Hospital, Riga, LVA

**Keywords:** antipsychotic therapy, breast imaging, core needle biopsy, diagnostic challenges, ductal carcinoma in situ, • galactorrhea, mammography, vacuum-assisted biopsy

## Abstract

Galactorrhea is most often associated with endocrine disorders or drug-induced hyperprolactinemia, particularly from antipsychotics. While commonly benign, persistent or unilateral galactorrhea with abnormal imaging findings warrants careful evaluation to exclude malignancy. We report the case of a 44-year-old woman receiving long-term antipsychotic therapy who developed unilateral (left-predominant) milky nipple discharge compatible with galactorrhea and breast tenderness. Mammography revealed diffusely distributed coarse heterogeneous calcifications, but two ultrasound-guided core needle biopsies (CNB) failed to identify malignancy, showing only papillomas with epithelial hyperplasia and atypia. Due to persistent radiologic suspicion, a tomosynthesis-guided vacuum-assisted biopsy (VAB) was performed and demonstrated low-grade ductal carcinoma in situ (DCIS). Mastectomy confirmed diffuse, multigrade DCIS (low-, intermediate-, and high-grade components), cribriform and comedo type, involving multiple breast quadrants. This case underscores the diagnostic challenges of attributing galactorrhea solely to medication side effects, highlights the limitations of core biopsy in diffuse calcifications, and emphasizes the importance of persistent diagnostic workup when imaging remains suspicious.

## Introduction

Galactorrhea unrelated to breastfeeding can result from pituitary adenomas, endocrine disorders, or, more commonly, drug-induced hyperprolactinemia, particularly in patients receiving antipsychotic medications. Antipsychotic medications, including dopamine-blocking agents such as risperidone and cariprazine, are well-known causes of hyperprolactinemia and galactorrhea [1-5]. Although drug-related galactorrhea is typically benign, unilateral nipple discharge, particularly when persistent or accompanied by abnormal imaging, requires careful evaluation, as symptoms may mask underlying malignant disease. Importantly, physiological galactorrhea caused by elevated prolactin is usually bilateral and is not associated with an increased risk of breast cancer, whereas pathological unilateral nipple discharge carries a significantly higher malignancy risk, reported in 3-23% of cases [6-9]. Because antipsychotic-induced galactorrhea results from systemic prolactin elevation, it is almost always bilateral; thus, a unilateral presentation is exceptionally rare and should prompt heightened concern for an underlying pathological process.

Ductal carcinoma in situ (DCIS) accounts for approximately 20% to 25% of screen-detected breast cancers and most commonly presents with calcifications rather than a palpable mass [10]. According to BI-RADS, calcification morphology is the primary determinant of malignancy risk, but distribution also provides important diagnostic information. DCIS most commonly presents as calcifications on mammography. While it typically manifests as grouped or segmental calcifications confined to a single ductal system, diffuse calcifications are more often associated with benign processes but do not exclude malignancy, particularly when the morphology is suspicious. Diffuse patterns may still conceal mixed benign, high-risk, and malignant epithelial changes, making diagnosis especially challenging when calcifications are extensive and multifocal [11]. 

Because diffuse calcifications may arise from a spectrum of benign, high-risk, and malignant epithelial changes, unilateral galactorrhea, although suggestive of a physiological drug effect, may obscure the early radiologic signs of DCIS and delay appropriate diagnostic escalation.

This report describes a patient whose unilateral galactorrhea, initially attributed to long-term antipsychotic medications, delayed recognition of extensive, multigrade DCIS. The case underscores the importance of maintaining diagnostic vigilance and obtaining targeted tissue sampling when imaging remains suspicious.

## Case presentation

Patient history and clinical findings

A 44-year-old woman presented with a 2-3-year history of intermittent discomfort and mild tenderness in the left breast, accompanied by inducible (non-spontaneous) white, milky nipple discharge that was more pronounced on the left. The milky character of the discharge was compatible with physiological galactorrhea; however, the unilateral predominance raised concern for an underlying pathological process. On physical examination, no palpable masses, skin changes, or axillary lymphadenopathy were detected. The patient reported no family history of breast or ovarian cancer, and no prior genetic testing had been performed. She was premenopausal at age 44.

Her medical history included childbirth nine years earlier and long-term psychotropic therapy for over five years, consisting of risperidone (Rispaxol® 2 mg daily), cariprazine (Reagila®), and trihexyphenidyl (Cyclodol® 2 mg daily) as an anticholinergic adjunct.

She had no prior screening mammograms for comparison. Because unilateral symptoms raised concern for a pathological cause, diagnostic imaging was performed. A comprehensive overview of the diagnostic pathway, including imaging, biopsies, and final surgical management, is summarized in Table 1.

**Table 1:** Chronological Timeline of Diagnostic Workup and Management Chronological summary of the patient’s clinical presentation, imaging findings, biopsy procedures, and management decisions. The table highlights progressive imaging–pathology discordance following two ultrasound-guided core needle biopsies, prompting escalation to tomosynthesis-guided vacuum-assisted biopsy and ultimately definitive surgical management. Final pathology confirmed extensive, multicentric ductal carcinoma in situ, underscoring the importance of correlating clinical features with imaging findings and escalating tissue sampling when discordance persists.

Time Point	Clinical / Imaging Event	Key Findings	Outcome / Next Step
~2–3 years prior	Onset of symptoms	Inducible milky nipple discharge; left-breast discomfort	Symptom progression; evaluation delayed
Day 0	First breast clinic visit; mammography, ultrasound, clinical exam	Mammography: diffuse coarse heterogeneous calcifications (BI-RADS 4); Ultrasound: dilated ducts, echogenic foci	Ultrasound-guided CNB (14G, 3 cores)
Day 0 – Biopsy 1	First ultrasound-guided CNB	Intraductal papillomas with epithelial hyperplasia; no atypia or malignancy	Imaging–pathology discordance → MDT recommends MRI
Day 7	Breast MRI	Diffuse asymmetric non-mass enhancement (BI-RADS 4)	Suspicion for DCIS persists → repeat biopsy
Day 14 – Biopsy 2	Second ultrasound-guided CNB (14G, 3 cores)	Papilloma with atypical ductal hyperplasia (ADH) — B3 lesion	Discordance persists → MDT recommends tomosynthesis-guided VAB
Day 21 – Biopsy 3	Tomosynthesis-guided VAB (9G)	Low-grade DCIS with associated ADH/FEA and calcifications	First malignant diagnosis → MDT recommends surgery
Day 28	Patient counseling	Reviewed extent, options, discordance	Patient declines further targeted sampling → elects definitive surgery
Day 40	Left mastectomy with immediate reconstruction	Extensive multicentric low-, intermediate-, and high-grade DCIS; mixed architectural patterns; clear margins	Confirms underestimation of disease on CNB; completes diagnostic pathway

Imaging

Mammography

**Figure 1:**
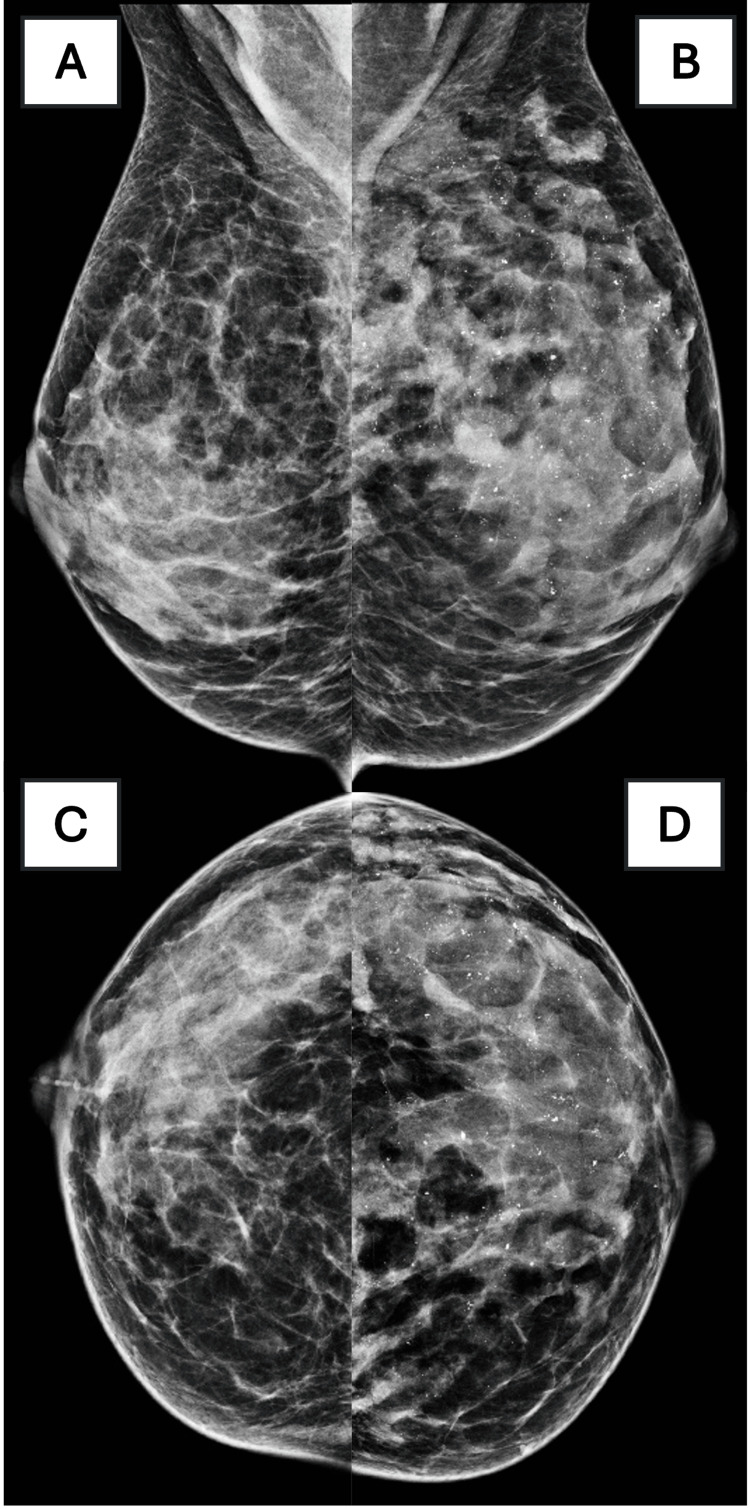
Initial Mammography (A) Right MLO. (B) Left MLO. (C) Right CC. (D) Left CC. The left breast shows diffuse coarse heterogeneous calcifications involving nearly the entire parenchyma, categorized as BI-RADS 4. Diffuse but suspicious calcifications prompted further diagnostic evaluation.

**Figure 2:**
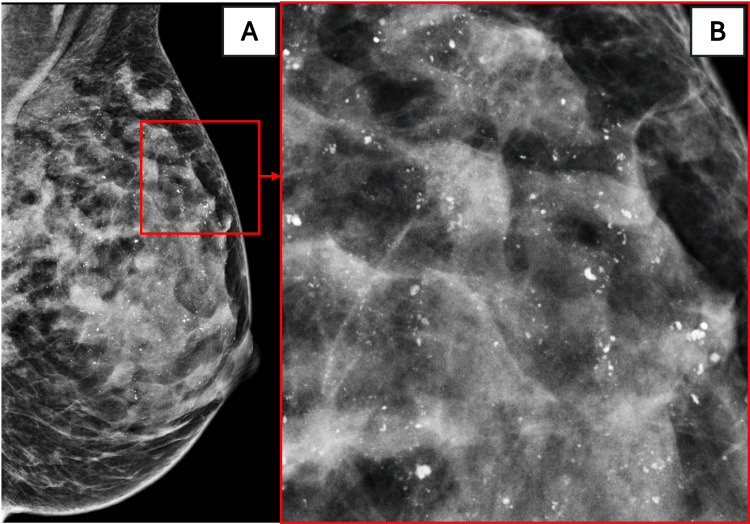
Magnification View (A) MLO view. (B) Magnified MLO view demonstrating coarse heterogeneous calcifications. *Magnification confirms suspicious morphology, helping guide targeted biopsy planning.*

Diagnostic workup started with mammography. Diagnostic digital mammography revealed diffusely distributed coarse heterogeneous calcifications in the left breast (BI-RADS 4; Breast Imaging Reporting & Data System “suspicious abnormality”) (Figure 1). Magnification views confirmed suspicious morphology (Figure 2).

Ultrasound

**Figure 3:**
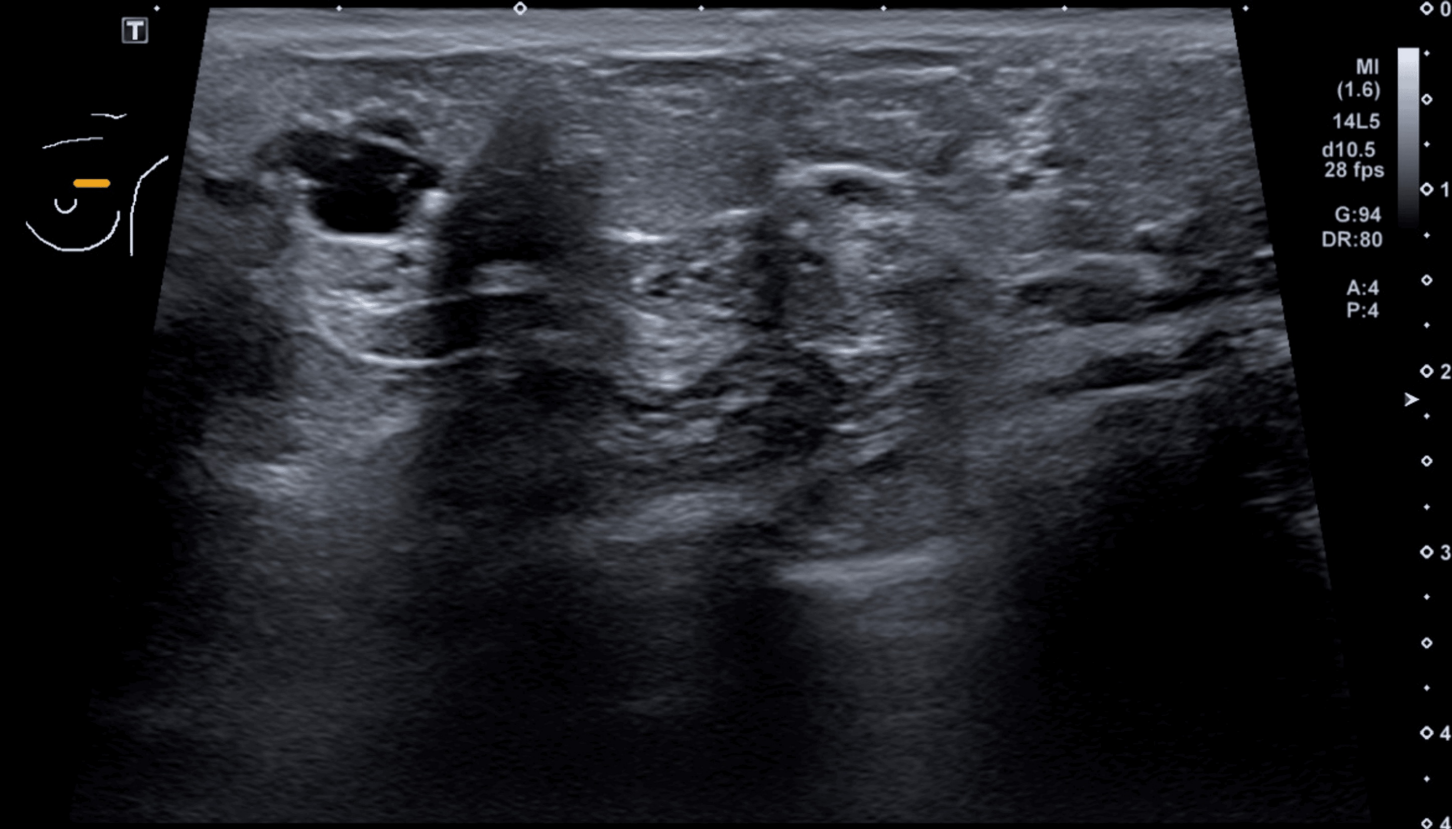
Diagnostic Breast Ultrasound Ultrasound demonstrates dilated ducts, fibrocystic changes, and hypoechoic foci containing echogenic calcifications correlating with mammography. A 14-gauge CNB was performed. Ultrasound correlation justified initial ultrasound-guided sampling.

Targeted ultrasound demonstrated dilated ducts, fibrocystic changes, and hypoechoic foci containing echogenic calcifications, corresponding to mammographic findings (Figure 3). No discrete mass was identified.

First Biopsy (Ultrasound-Guided Core Needle Biopsy (CNB)

A 14-gauge ultrasound-guided CNB (three cores) was performed targeting the most conspicuous area. Histopathology showed intraductal papillomas with epithelial hyperplasia, without atypia or malignancy. Because diffuse calcifications can be undersampled with limited cores, and due to imaging-pathology discordance, further evaluation was pursued.

MRI Evaluation

**Figure 4:**
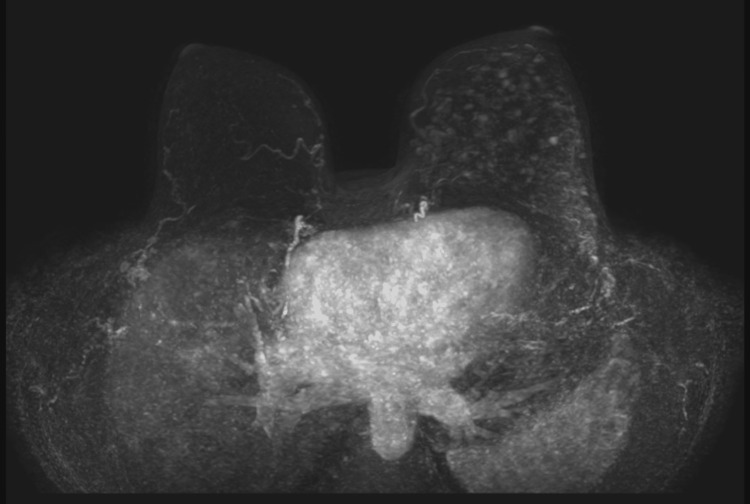
Problem-Solving Breast MRI Dynamic contrast-enhanced MRI (subtraction MIP) shows diffuse asymmetric non-mass enhancement correlating with mammographic abnormalities (BI-RADS 4). *The extent of non-mass enhancement increased suspicion for widespread DCIS and supported the need for further tissue sampling.*

Problem-solving dynamic contrast-enhanced MRI demonstrated diffuse non-mass enhancement asymmetrically involving the left breast (BI-RADS 4), consistent with widespread ductal abnormality (Figure 4). Enhancement characteristics raised suspicion for extensive DCIS.

Second Biopsy (Ultrasound-Guided CNB)

A repeat ultrasound-guided biopsy targeted a more posterior region. This biopsy revealed papillomas with atypical ductal hyperplasia (ADH), a B3 lesion (lesion of uncertain malignant potential), again insufficient to explain the extensive calcifications and MRI findings.

Third Biopsy (Tomosynthesis-Guided Vacuum-Assisted Biopsy)

**Figure 5:**
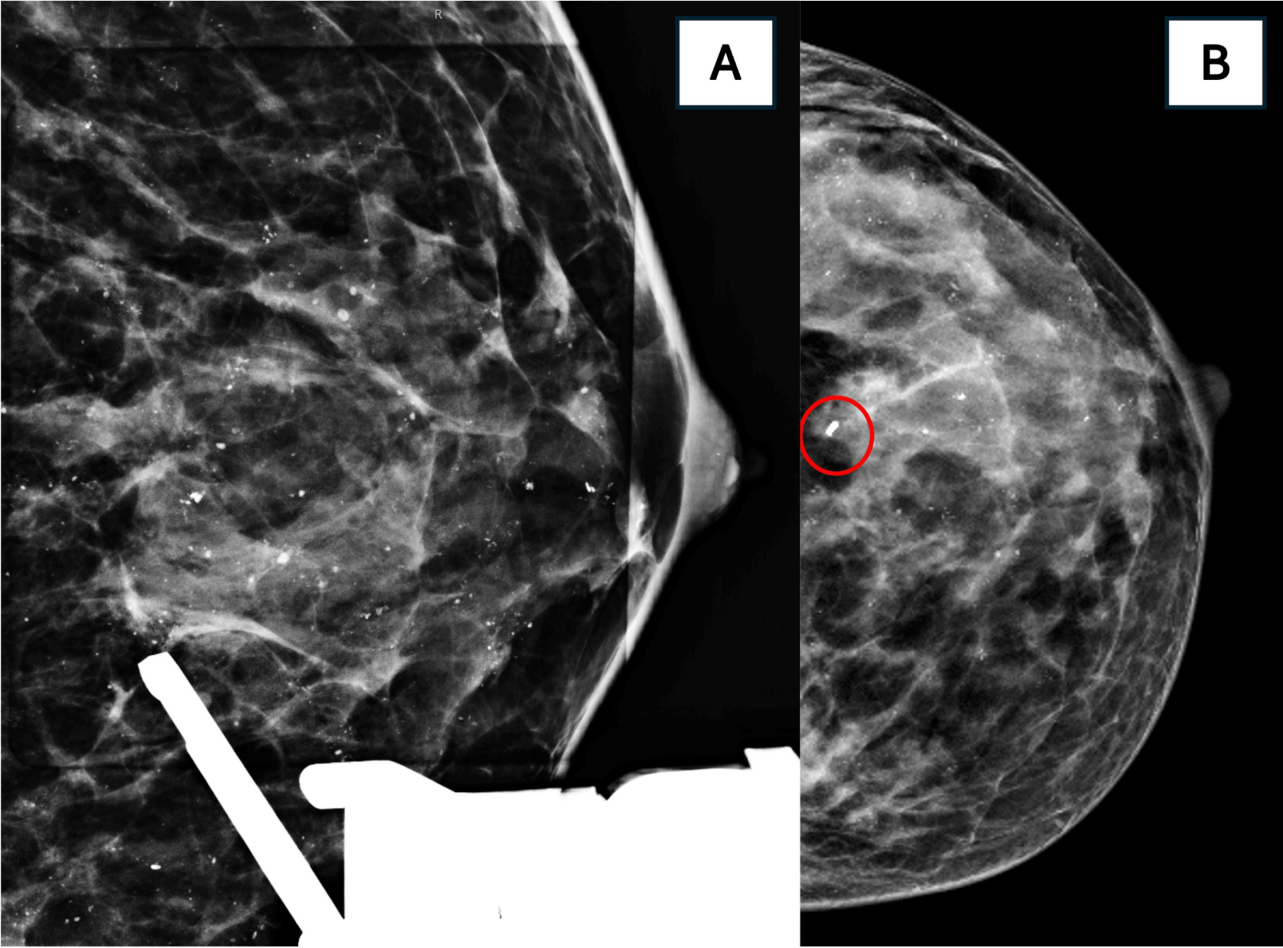
Tomosynthesis-Guided Vacuum-Assisted Biopsy (A) Stereotactic targeting of suspicious calcifications using 9-gauge VAB. (B) Post-biopsy CC view confirming clip placement (red circle). Histopathology revealed low-grade DCIS with ADH, flat epithelial atypia, and calcifications. *Tomosynthesis guidance enabled accurate retrieval of calcifications after two discordant CNBs.*

Because of persistent discordance, a 9-gauge tomosynthesis-guided vacuum-assisted biopsy (VAB) sampled the calcifications directly (Figure 5). This yielded multifocal low-grade ductal carcinoma in situ (DCIS) with a micropapillary pattern, associated flat epithelial atypia, ADH, and calcifications. No comedo-type necrosis was present. HER2 immunohistochemistry was negative; estrogen receptors (ER) were 90%, progesterone receptor (PR) 70%, and the Ki-67 index 5%.

Endocrine evaluation

Laboratory tests showed mildly elevated prolactin levels of 36 ng/mL (reference < 25 ng/mL), consistent with psychotropic-associated hyperprolactinemia. Thyroid-stimulating hormone levels were normal. Brain MRI showed no pituitary adenoma.

Surgical management

The patient declined additional targeted sampling of other suspicious quadrants and opted for definitive treatment. She underwent a left mastectomy with immediate reconstruction.

**Figure 6:**
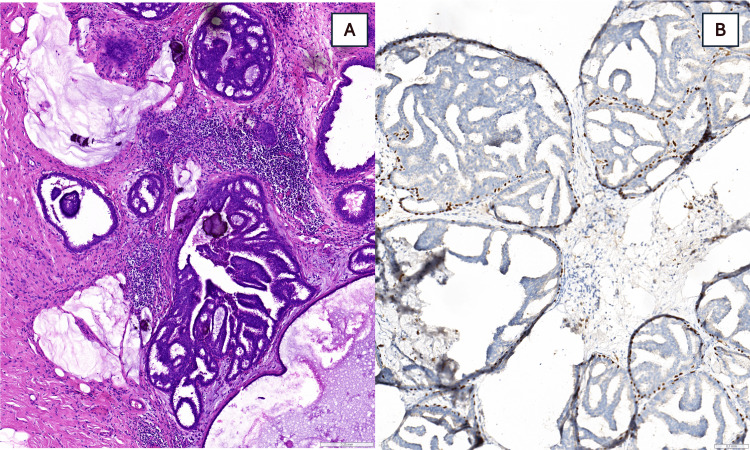
Histopathology and Immunohistochemistry From Mastectomy Specimen (A) Low- and intermediate-grade DCIS (cribriform and micropapillary patterns) with calcifications, ADH, flat epithelial atypia, and stromal fibrosis. High-grade DCIS was present elsewhere but not shown in this section. (B) p63 immunohistochemistry highlights the myoepithelial layer surrounding the involved ducts. *These findings illustrate the heterogeneous, multicentric nature of the disease, explaining why earlier CNBs under-sampled the malignant component.*

Final surgical pathology confirmed extensive, multicentric low-, intermediate-, and high-grade DCIS, cribriform, micropapillary, and comedo type (characterized by central necrosis within ducts), with microcalcifications and focal flat epithelial atypia involving multiple quadrants. In the surrounding areas, there were fibrocystic changes with multiple papillomas, luminal cell hyperplasia, and apocrine metaplasia (Figure 6). Because the disease was diffuse and involved several quadrants without a single dominant focus, a discrete tumor size could not be reported; in such cases, extent is most accurately described by distribution rather than linear measurement. There were no residual tumor cells at the resection margins, and the closest margin was 2 mm. The ducts contained residual white, milk-like fluid, consistent with galactorrhea. Lymph nodes were not involved.

## Discussion

This case underscores several important clinical and radiological lessons.

Clinical implications of drug-induced galactorrhea

Antipsychotic-associated galactorrhea is common and is typically bilateral, inducible, and benign, which may lower clinical suspicion for underlying breast pathology [4,12]. In our patient, the clear left-sided predominance represented an atypical feature that warranted heightened concern despite a plausible pharmacologic explanation. The patient demonstrated only mild prolactin elevation (36 ng/mL), a range commonly reported in drug-induced hyperprolactinemia and sufficient to produce galactorrhea in susceptible individuals. However, existing literature indicates that prolactin level alone does not reliably distinguish benign from pathological nipple discharge, and unilateral discharge, irrespective of prolactin level, carries a substantially higher malignancy risk, reported between 3% and 23% [7-9,13].

This case illustrates how a well-recognized medication side effect can contribute to diagnostic anchoring bias, lowering clinical suspicion despite concurrent suspicious imaging findings. Similar pitfalls have been described in nipple discharge evaluation, where plausible benign explanations delayed escalation of tissue sampling [6,7]. Our findings reinforce that imaging-pathology concordance should remain the primary driver of management, rather than symptom etiology alone.

Rather than challenging the benign nature of antipsychotic-induced galactorrhea itself, this case highlights the need for continued diagnostic vigilance when unilateral symptoms coexist with abnormal mammographic or MRI findings.

Diagnostic challenges in diffuse breast calcifications on mammograms

When evaluating calcifications, both morphology and distribution must be considered. A diffuse distribution is more often associated with benign processes than with malignancy. In this case, the calcifications in the left breast were categorized as diffusely distributed coarse heterogeneous calcifications, for which the reported malignancy risk ranges from approximately 7% to 21%.

These calcifications may correspond to a spectrum of epithelial alterations, including benign hyperplasia without atypia, papillomatosis with or without atypia, usual ductal hyperplasia, atypical ductal hyperplasia (ADH), flat epithelial atypia (FEA), and widespread low- or intermediate-grade ductal carcinoma in situ (DCIS). These entities may coexist within diffuse epithelial proliferation, making limited sampling particularly prone to false-negative results. The initial two 14-gauge CNB procedures (three cores each), therefore, likely failed to capture the malignant component. Compared with CNB, VAB shows better diagnostic performance in terms of DCIS underestimation and microcalcification retrieval rate.

Although diffuse papillomatosis with or without ADH, found on the second CNB, can occasionally explain diffuse calcifications and may be associated with nipple discharge, the overall clinical, imaging, and histologic findings remained discordant. The milky discharge was more suggestive of physiological galactorrhea rather than the typically serous or serosanguineous discharge seen with papillomas. Moreover, ADH is a B3 lesion, and European guidelines recommend VAB as the next diagnostic step to exclude coexistent DCIS [14]. In diffuse disease, it is always challenging to determine which region should be targeted for biopsy; in this case, the radiologist selected the area with the highest level of suspicion.

Recent consensus guidelines support the use of ultrasound-guided biopsy as the first choice for calcifications that are visible on ultrasound because ultrasound guidance is widely available, minimally burdensome, and allows real-time targeting [15]. Accordingly, we initially performed ultrasound-guided CNB. However, VAB provides substantially higher diagnostic accuracy than CNB, particularly for calcifications. A large meta-analysis reported that DCIS underestimation was significantly lower with VAB (11.05%) compared with CNB (22.98%), and microcalcification retrieval was markedly higher with VAB (98.3% vs. 83.1%) [16]. EUSOBI recommends vacuum-assisted biopsy as the preferred technique for sampling microcalcifications, particularly when they are diffuse, extensive, or only visible on mammography or tomosynthesis. Tomosynthesis-guided VAB, especially with a 9-gauge device, provides superior calcification retrieval and reduces DCIS underestimation by enabling acquisition of larger, more contiguous tissue volumes [17]. 

Importance of multidisciplinary evaluation

The final diagnosis was achieved only after coordinated review between radiologists, pathologists, surgeons, and endocrinologists. This case reinforces that discordant findings require escalation, including repeat or alternative biopsy methods, until a unifying diagnosis is obtained.

## Conclusions

Unilateral galactorrhea in patients receiving antipsychotic therapy should not be attributed solely to medication, particularly when imaging demonstrates diffuse suspicious calcifications. Antipsychotic-induced galactorrhea is typically bilateral, making a unilateral presentation extremely rare, and should heighten suspicion for underlying breast pathology. Physiologically, prolactin-related galactorrhea is generally bilateral and non-spontaneous and therefore does not exclude malignancy when symptoms are asymmetric. Clinicians should maintain vigilance and pursue escalated sampling when unilateral symptoms persist despite benign initial biopsy results. This case highlights the limitations of core needle biopsy in diffuse calcifications, the diagnostic value of tomosynthesis-guided vacuum-assisted biopsy, and the importance of multidisciplinary review when imaging and pathology are discordant. Although tomosynthesis-guided VAB successfully retrieved calcifications, it sampled only low-grade DCIS with precursor lesions; the full extent of disease, including low-, intermediate-, and high-grade DCIS, became apparent only in the mastectomy specimen. This discrepancy underscores the inherent sampling limitations of biopsy techniques in diffuse, multifocal processes and reinforces the need for persistent diagnostic escalation when imaging suspicion remains high.

## References

[1] Gosi SKY, Garla VV (2025). Galactorrhea. https://www.ncbi.nlm.nih.gov/books/NBK537115/.

[2] Bruehlman RD, Winters S, McKittrick C (2022). Galactorrhea: rapid evidence review. Am Fam Physician.

[3] Vilar L, Vilar CF, Lyra R, Freitas MD (2019). Pitfalls in the diagnostic evaluation of hyperprolactinemia. Neuroendocrinology.

[4] Stojkovic M, Radmanovic B, Jovanovic M (2022). Risperidone induced hyperprolactinemia: from basic to clinical studies. Front Psychiatry.

[5] Hope J, Keks NA (2022). Cariprazine: a new partial dopamine agonist with a familiar profile. Austr Psych.

[6] Pitarch M, Alcantara R, Comerma L (2025). An update on multimodal imaging strategies for nipple discharge: from detection to decision. Insights Imaging.

[7] Clark SE, Agrawal A, Laws S (2020). The investigation and management of unilateral nipple discharge. Ann R Coll Surg Engl.

[8] Chen L, Zhou WB, Zhao Y (2012). Bloody nipple discharge is a predictor of breast cancer risk: a meta-analysis. Breast Cancer Res Treat.

[9] Morrogh M, Park A, Elkin EB, King TA (2010). Lessons learned from 416 cases of nipple discharge of the breast. Am J Surg.

[10] Barnes NL, Dimopoulos N, Williams KE (2014). The frequency of presentation and clinico-pathological characteristics of symptomatic versus screen detected ductal carcinoma in situ of the breast. Eur J Surg Oncol.

[11] American College of Radiology (2025). American College of Radiology: BI-RADS: ACR breast imaging reporting and data system. https://www.acr.org/Clinical-Resources/Clinical-Tools-and-Reference/Reporting-and-Data-Systems/BI-RADS.

[12] Chanson P (2022). Treatments of psychiatric disorders, hyperprolactinemia and dopamine agonists. Best Pract Res Clin Endocrinol Metab.

[13] Peña KS, Rosenfeld JA (2001). Evaluation and treatment of galactorrhea. Am Fam Physician.

[14] Rubio IT, Wyld L, Marotti L (2024). European guidelines for the diagnosis, treatment and follow-up of breast lesions with uncertain malignant potential (B3 lesions) developed jointly by EUSOMA, EUSOBI, ESP (BWG) and ESSO. Eur J Surg Oncol.

[15] Sanderink WB, Camps-Herrero J, Athanasiou A (2025). Image-guided biopsy of breast lesions-when to use what biopsy technique. Insights Imaging.

[16] Huang XC, Hu XH, Wang XR (2018). A comparison of diagnostic performance of vacuum-assisted biopsy and core needle biopsy for breast microcalcification: a systematic review and meta-analysis. Ir J Med Sci.

[17] Bick U, Trimboli RM, Athanasiou A (2020). Image-guided breast biopsy and localisation: recommendations for information to women and referring physicians by the European Society of Breast Imaging. Insights Imaging.

